# Evidence of a distinct group of Black African patients with systemic lupus erythematosus

**DOI:** 10.1136/bmjgh-2017-000697

**Published:** 2018-09-16

**Authors:** Elopy N Sibanda, Margo Chase-Topping, Lorraine T Pfavayi, Mark E J Woolhouse, Francisca Mutapi

**Affiliations:** 1 Asthma, Allergy and Immunology Clinic, Twin Palms Medical Centre, Harare, Zimbabwe; 2 TIBA Partnership, NIHR Global Health Research Unit Tackling Infections to Benefit Africa (TIBA), University of Edinburgh, Edinburgh, UK; 3 Centre for Immunity, Infection and Evolution, School of Biological Sciences, University of Edinburgh, Edinburgh, UK; 4 Usher Institute of Population Health Sciences and Informatics and Centre for Immunity, Infection and Evolution, School of Biological Sciences, University of Edinburgh, Edinburgh, UK; 5 Institute of Immunology and Infection Research, Centre for Immunity, Infection and Evolution, School of Biological Sciences, Ashworth Laboratories, University of Edinburgh, Edinburgh, UK

**Keywords:** elisa, serology, screening, public health

## Abstract

**Background:**

The autoimmune disease systemic lupus erythematosus (SLE) occurs more frequently in patients of African descent with high morbidity and mortality. Current SLE diagnostic criteria including antinuclear antibody (ANA) reactivity are derived largely from non-African populations. This study characterises ANA reactivity patterns and relates them to SLE clinical presentation in Black African patients.

**Methods:**

Sera from Black participants (61 patients with SLE and 100 controls) aged 1–81 years were analysed for reactivity against the antigens: uridine 1-ribonuclear protein, Smith uridine-1-5 ribonuclear protein antigen, soluble substance-A, recombinant Ro-52, soluble substance-B, Scl-70, cytoplasmic histidyl-tRNA synthetase antigen, proliferating cell nuclear antigen (PCNA), nucleosomes, ribonuclear P-protein, antimitochondrial antibody M2 (AMA-M2), histones, double-stranded DNA (dsDNA), centromere protein B and polymyositis–sclerosis overlap antigen.

**Findings:**

A significantly higher proportion (97%) of the 61 patients with SLE had detectable autoantibody reactivity compared with 15% of the 100 controls (p<0.001). The highest frequencies of autoantibody reactivity in patients with SLE were against the dsDNA antigen (41%) and PCNA (54%). Anti-PCNA and anti-dsDNA reactivity were mutually exclusive (p<0.001) giving rise to two distinct groups of Black African patients with SLE. The first group (n=25) had reactivity profiles consistent with international standard SLE definitions, including anti-dsDNA reactivity, and was 13 times more likely to present with joint symptoms. The larger, second group (n=34), characterised by anti-PCNA and anti-AMA-M2 reactivity, was nine times more likely to present with only cutaneous symptoms.

**Interpretation:**

Our study demonstrates a need to extend autoantibody panels to include anti-PCNA in the diagnostic process of Black African patients and further refine the predictive values of the reactivity to different antigens to differentiate SLE syndromes in African populations.

**Key questions**

**What is already known?**

- Systemic lupus erythematosus (SLE) occurs more frequently in patients of African descent but the scientific basis for SLE diagnostic criteria is derived from non-African populations.
- Refining the predictive values of antinulcear antibody subtype reactivity to different nuclear antigens will aid differentiation of SLE syndromes in African populations.

**What are the new findings?**

- Our key finding is that there exists a large subgroup (54%) of Southern African patients whose laboratory tests differ from the international American College of Rheumatology Classification diagnostic guidelines for SLE.
- This group did not react against dsDNA, but rather were reactive against proliferating cell nuclear antigen (PCNA) and was nine times more likely to present with only cutaneous symptoms.

**What do the new findings imply?**

- Our findings show that there is a need to consider the two antinuclear antigens, PCNA and antimitochondrial antibody M2, as additional diagnostic markers for patients with SLE.

## Introduction

Systemic lupus erythematosus (SLE) is a complex, chronic autoimmune disease that can affect multiple organs including the skin, joints, haematopoietic system, kidneys, lungs and the central nervous system.^1^ Previous studies report differences in the prevalence and severity of SLE in different ethnic groups.^2^ SLE-associated mortality in Black American patients is 24% compared with 5% among Asians with comparable demographic and clinical features.^3^ SLE diagnosis in Africa remains challenging and true disease and mortality rates are unknown.^4^ Treatment is often complicated by side effects,^5^ so accurate, and early diagnosis (which can be facilitated by characterising autoantibody reactivity) is key to successful disease management.^6^ Current SLE diagnostic criteria were defined through collaborations including the American Rheumatism Association (ARA)^7 8^ and the Systemic Lupus International Collaborating Clinics Classification (SLICC)^9^ whose criteria are now referred to as the American College of Rheumatology (ACR) criteria.^7 8^ Even though SLE occurs more frequently in patients of African descent,^10^ the scientific basis for SLE classification is derived predominantly from studies in non-African populations. The revised criteria for SLE classification includes detection of antinuclear antibodies (ANA) and extractable nuclear antibodies (ENA)^11^ which mediate the disease and are associated with distinct SLE disease subsets and progression.^12 13^ Autoantibody production is influenced by, among other factors, human leucocyte antigen (HLA) haplotype,^14^ with the HLA haplotype believed to influence disease prevalence, for example, increased rates of SLE in African-Americans and more frequent detection of SLE in first-degree relatives^15 16^compared with unrelated people. The biomarkers of SLE may differ in different racial populations, for example, patients of African ancestry are reportedly more likely to have anti-Sm antibodies compared with those of European ancestry.^17^

Studies validating the SLICC SLE diagnostic criteria (eg, Petri *et al* 9) have not included Black patients resident in Africa. In addition, the epidemiology of SLE in Africa remains largely unknown. The disease is underreported in Africa due to several reasons that include poor access to healthcare, low disease recognition within primary healthcare settings, limited access to diagnostic tools and inadequate numbers of relevant specialists.^4^ To help address some of these issues, we have conducted this study to inform SLE diagnosis in Africa through characterisation of autoantibody reactivity profiles in Black African patients with SLE and relating their autoantibody reactivity to clinical symptoms.

## Methods

### Patients and controls

This retrospective study consisted of 161 participants aged 1–81 years. All patients had been referred to the Asthma, Allergy and Immune Dysfunction Clinic in Harare, Zimbabwe, for diagnosis and management of suspected allergic, immunodeficiency or autoimmune conditions during the period between July 2010 and April 2014. Ethical approval for the study was obtained from the Medical Research Council of Zimbabwe and written informed consent to use results of clinical assessment for research was obtained from the participants/guardians as appropriate. All participants were permanent residents in Zimbabwe and they self-reported ethnicity in terms of race as Black, White, Asian or mixed race. Given the sample size available for the other ethnicities, only Black African participants were included in the study. The clinician (ES) conducted case history collation and clinical examination of all participants. For inclusion into the study, participants had to be negative for HIV infection and be permanent residents of Zimbabwe. Data were anonymised through assignment of unique identification numbers for each patient prior to transferring for analysis. Of the 161 participants, 61 who fulfilled the ACR criteria^8^ were diagnosed as positive for SLE.

The 100 controls represent patients who were negative for any autoimmune disease or allergic disease. These patients had been referred to the clinic for investigation of allergy and were tested and clinically evaluated for both allergy and autoimmune diseases and were found negative for both. They were tested at the same period as the SLE cases but have a slightly larger range of ages (1–81 vs 2–67 years for cases). To ensure that no results were attributable to differences in gender or age range, we carried out two further analyses using age-matched and gender-matched patients with SLE and controls at both a 1:1 and 2:1 ratio of controls to patients and calculated the proportions reacting to the different nuclear and mitochondrial antigens. The results were consistent across all analyses, and therefore, 61 SLE cases and 100 controls were included in the study. The gender and sex distributions of the participants are given in table 1.

**Table 1:** Study population

Clinical diagnosis	N	Median age (years)	Minimum age (years)	Maximum age (years)
Controls				
Female	70	28.0	1	81
Male	30	14.5	1	79
Total	100	27.0*	1	81
SLE				
Female	42	30.5	2	67
Male	19	38.0	2	52
Total	61	34.0*	2	67

*Statistical comparison of the distribution of ages between the patients with SLE and the controls showed no significant difference (Mann-Whitney test, p=0.240).

SLE, systemic lupus erythematosus.

### Clinical and laboratory assessment

**Figure 1:**
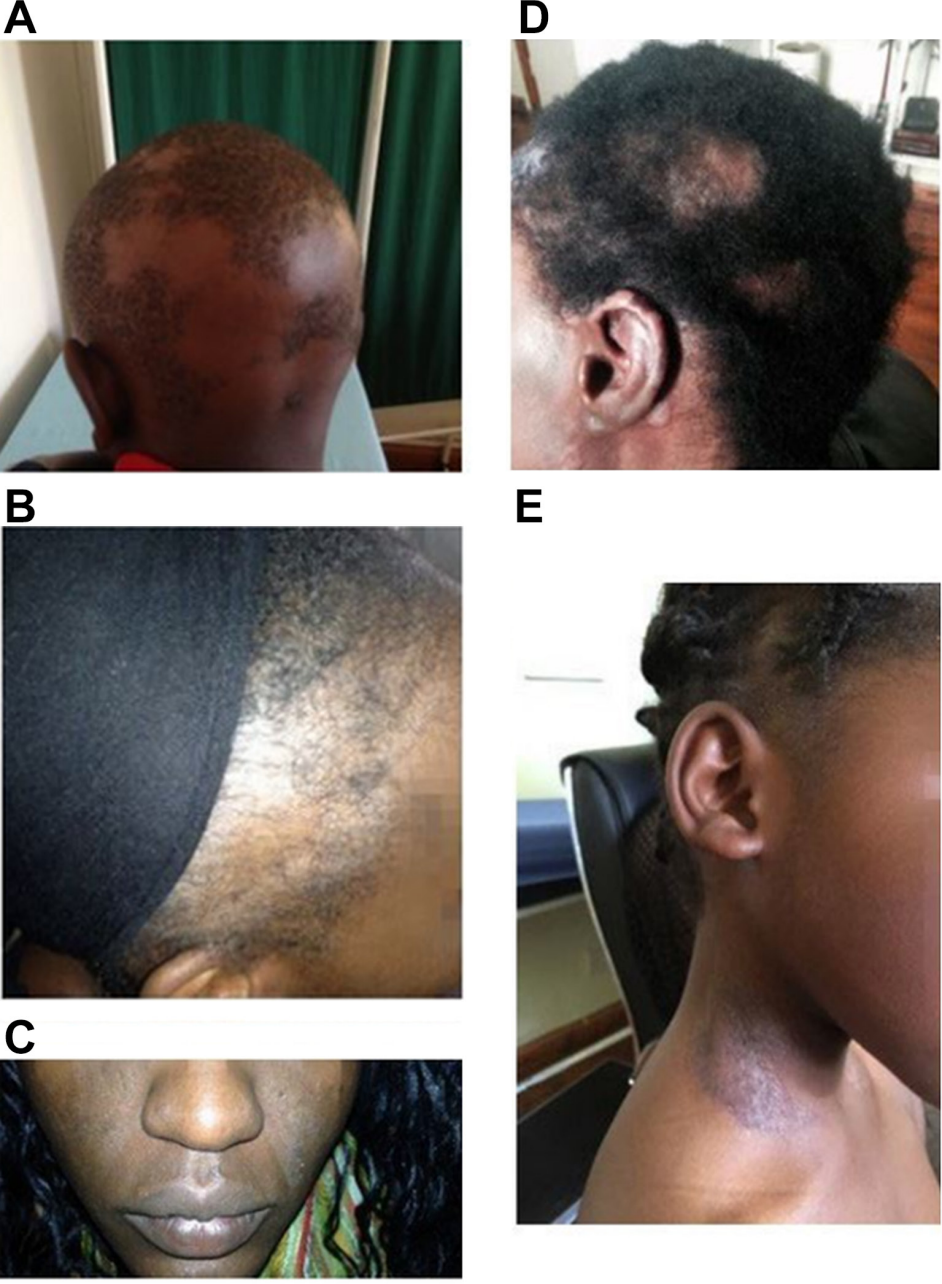
Photographs of patients’ physical symptoms. (A) Non-scarring, non-atrophic patchy hair loss, alopaecia areata in a 14-year-old boy with SLE (dsDNA+). (B) Diffuse thinning and loss of hair in a 45-year-old woman (dsDNA+). (C) A 36-year-old woman with a rash involving the malar area and nose, sparing the naso-labial folds (SLE butterfly rash) (dsDNA−; PCNA−). (D) A 54-year-old woman with scaring alopaecia of the scalp, and scaring lesions on the ear lobes and parts of the face (PCNA+). (E) A 13-year-old girl with non-scarring photosensitive dermatitis of the neck and face (PCNA+). dsDNA, double-stranded DNA; PCNA, roliferating cell nuclear antigen; SLE, systemic lupus erythematosus.

A diagnosis of SLE was made based on the patient’s clinical history, symptoms at presentation (eg, figure 1) and relevant laboratory investigations. All participants fulfilling the ACR criteria for SLE were categorised as positive for SLE. Analysis for sero-reactivity against the nuclear and mitochondrial antigens was conducted for all 61 patients with SLE and the 100 controls. In 52 of the patients with SLE, the disease stage did not require repeated evaluation thus autoantibody reactivity was assessed once. All these patients were offered routine clinical monitoring to guide laboratory investigations. The remaining nine patients with SLE were followed up at least twice in 4 years. While asthma and rhinitis symptoms are not features of SLE, the conditions are not mutually exclusive and some of the patients with SLE presented with these comorbidities. These and any other comorbidities were recorded and included in the statistical analyses. Laboratory tests were guided by clinical presentation and medical history. These included haematology, biochemistry, serology and immunology, (immunoglobulin (Ig)A, IgM and IgG reactivity to a panel of nuclear antigens, complement (C3 and C4)), erythrocyte sedimentation rate, C reactive protein, cardiolipin and glycoprotein determination.

### Autoantibody reactivity determination

Patients’ serum samples were analysed using a commercially available ANA Profile 3 Euroline Cat # DL 1590-6401-3 G immunoblot kit containing the 15 autoantigens listed in table 2, following the manufacturer’s instructions. This kit has previously been used successfully either together with conventional indirect immunofluorescence for comparison, or alone to characterise connective tissue or autoimmune diseases.^18–21^ Briefly, immunoblot test strips impregnated with 15 antigens were incubated with sera diluted at 1:101 in a sample buffer. In reactive samples, serum antibodies bind to their respective autoantigenic sites. The bound autoantibodies are detected by incubating the strips with alkaline phosphatase labelled anti-human IgG antisera. The addition of the substrate elicits a colour reaction evaluated visually and semiquantitatively using the manufacturer’s software (EUROlabScan) to indicate positive or negative reactivity. A control band is included in each strip.

**Table 2:** Antinuclear antigens on the diagnostic test strip

Antigen	Immunoblot assay kit manufacture’s detail
nRNP/Sm	Uridine 1-ribonuclear protein (U1-nRN) purified by affinity chromatography from calf and rabbit thymus
Sm	Smith uridine-1-5 ribonuclear protein antigen purified by affinity chromatography from bovine spleen and thymus. The Sm antigen contains the core proteins of snRNP particles. D protein is the main component of the Sm preparation
SS-A	Soluble substance-A (60 kDa) purified by affinity chromatography from bovine spleen and thymus
Ro-52	Recombinant Ro-52 (52 kDa). The corresponding human cDNA has been expressed with the baculovirus system in insect cells
SS-B	Soluble substance-B antigen purified by affinity chromatography from calf and rabbit thymus
Scl-70	DNA topoisomerase antigen purified by affinity chromatography from bovine and rabbit thymus
PM-Scl	Polymyositis–sclerosis overlap antigen. Recombinant PM-Scl100 whose corresponding human cDNA has been expressed with the baculovirus system in insect cells
Jo-1	Cytoplasmic histidyl-tRNA synthetase antigen purified by affinity chromatography from calf and rabbit thymus
CENP-B	Recombinant centromere protein B whose corresponding human cDNA has been expressed with the baculovirus system in insect cells
PCNA	Proliferating cell nuclear antigen. Recombinant PCNA (36 kDa) whose corresponding human cDNA has been expressed with the baculovirus system in insect cells
dsDNA	Double-stranded DNA. The highly purified native, double-stranded DNA was isolated from salmon testes
Nucleosomes	Native nucleosomes purified from calf thymus
Histones	A mixture of individually purified histone types isolated from calf thymus
Rib. P-protein	Ribosomal P proteins purified by affinity chromatography
AMA-M2	Purified antimitochondrial M2 (pyruvate–dehydrogenase complex) antigen

### Statistical analysis

Reactivity results for the 15 autoantigens were recorded as presence or absence. Test results from (n=11) patients with follow-up visits (from 2 to 4) were combined for the purposes of analysis: auto antibodies against the antigen were considered present if detected during at least one visit. The frequency of autoantibody reactivity for patients with SLE versus controls was compared using the Fisher’s exact test. The frequency of reactivity was <5% against CENP-B, Jo-1, PM-Scl, Scl-70, nRNP/Sm and SS-B and >5% for SS-A, Sm, Ro-52, PCNA, nucleosomes, Rib. P-protein, histones, dsDNA and AMA-M2. Associations across all antigens (except anti-CENP-B where no patients were reactive) were examined using tetrachoric correlations (Proc freq, SAS V.9.4), (online supplementary table S1).

10.1136/bmjgh-2017-000697.supp1 Supplementary data

Autoantibody reactivity was further characterised in the patients with SLE using two-way cluster analysis (PC ORD V.6.08^22^)employing Jaccard distance and Group average method to generate clusters. Two out of the 61 patients were not included in the analyses, as they were not reactive to any of the antigens. The number of significant clusters was determined by creating different levels of clustering (n=2–8 clusters) within the dendogram and plotting the distance between each cluster.^12^ The number of clusters was determined by breakpoint analysis. Thereafter, cluster designation was used to determine associations with clinical symptoms using either Fisher’s exact test or Χ^2^ analysis depending on the structure of the data. Clinical symptoms from patients were partitioned into five summary groups: cutaneous, joint, asthma and rhinitis-like, gastrointestinal and other (online supplementary table S2). Statistical significance was set a priori at p<0.05. To check whether there may be subgroups within clusters, the two clusters obtained were divided into smaller groups and the association tests run to determine if the groups had different associations with the clinical signs. There were no significant differences within the main clusters with respect to the association with clinical signs and therefore, the two clusters were maintained.

10.1136/bmjgh-2017-000697.supp2 Supplementary data

The frequency of autoantibody reactivity among patients with SLE was compared with reference frequencies published from other populations^11 23–26^ and from test populations used for validating the diagnostic kit by the kit manufacturer (https://www.euroimmun.com/?id=2589).

## Results

### Autoantibody reactivity profile

**Figure 2:**
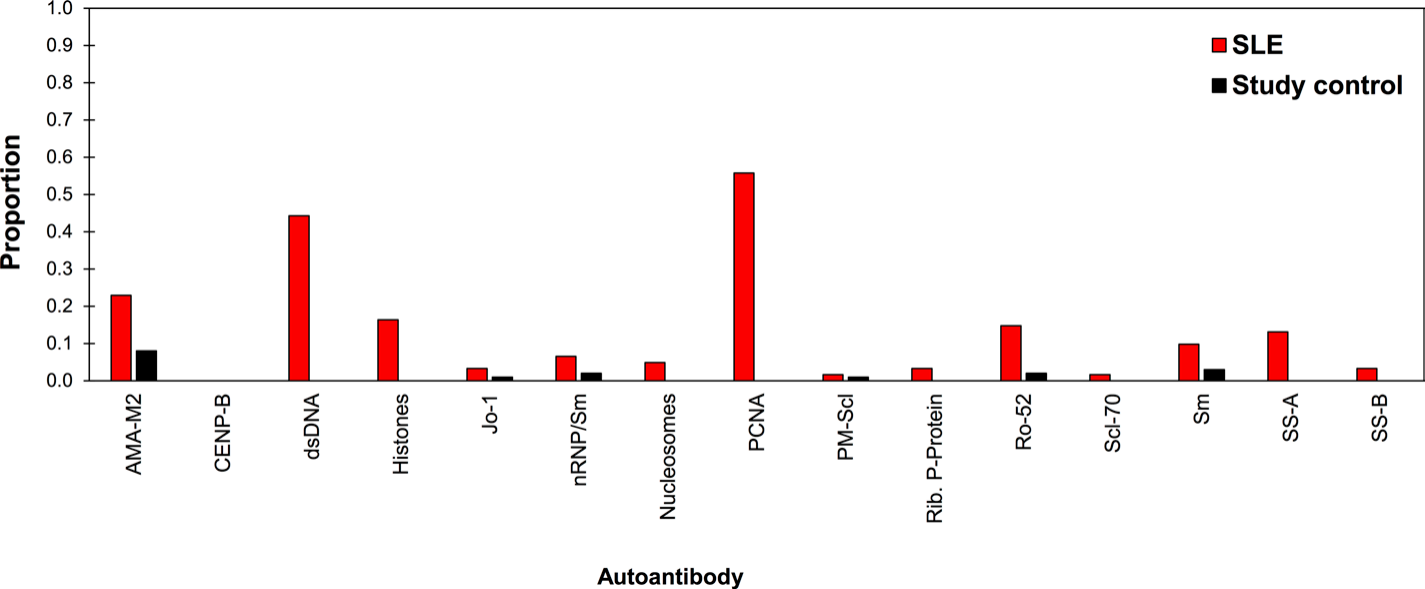
Proportion of participants diagnosed with SLE (n=61) and study control patients (n=100) who are reactive against each antinuclear antigens. AMA-M2, antimitochondrial antigen M2; CENP-B, centromere protein B; dsDNA, double-stranded DNA; Jo-1, cytoplasmic histidyl-tRNA synthetase antigen; nRNP/Sm, uridine 1-ribonuclear protein; PCNA, proliferating cell nuclear antigen; PM-Scl, polymyositis–sclerosis overlap antigen; Rib. P-protein, ribosomal P protein; Ro-52, recombinant Ro-52; SLE, systemic lupus erythematosus; Sm, Smith uridine-1-5 ribonuclear protein antigen; SS-A, soluble substance-A; SS-B, soluble substance-B.

A significantly higher proportion (97%) of the 61 patients with SLE were autoantibody reactive compared with 15% of the 100 controls (Fisher’s exact=123, df=1, p<0.001). The autoantibodies detected in the 15% control participants were directed against one or more of the nuclear antigens nRNP/Sm, Sm, SS-A, Ro-52, Jo-1, PM-Scl and AMA-M2, with frequencies predominantly <5% (figure 2, online supplementary table S3). The highest frequencies of autoantibody reactivity in patients with SLE were against dsDNA (41%) and PCNA (54%). Two patients who fulfilled the ACR clinical criteria for SLE diagnosis showed no autoantibody reactivity.

10.1136/bmjgh-2017-000697.supp3 Supplementary data

### Correlations between autoantibody reactivity

**Figure 3:**
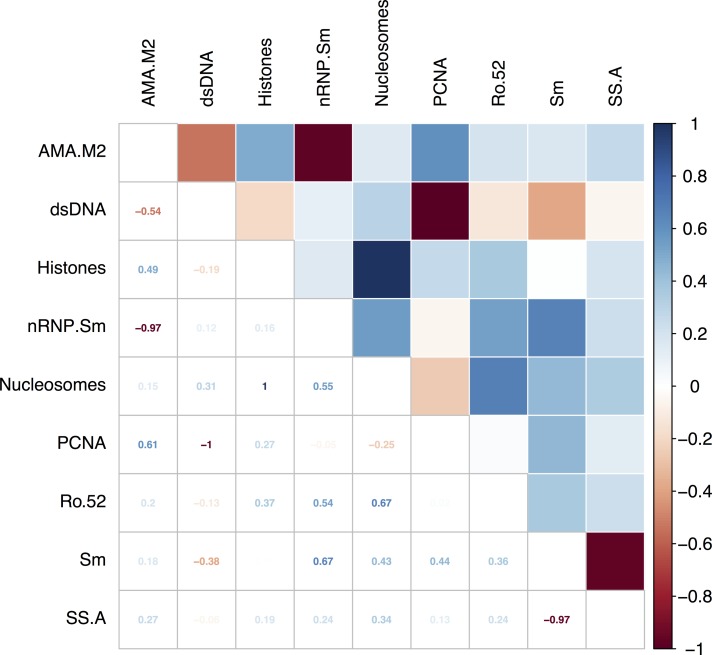
Figure showing the tetrachoric correlation coefficients for nine dichotomous autoantibodies with reactivity frequency >5% among the patients with SLE (n=61). AMA-M2, antimitochondrial antigen M2; dsDNA, double-stranded DNA; nRNP/Sm, uridine 1-ribonuclear protein; PCNA, proliferating cell nuclear antigen; Ro-52, recombinant Ro-52; SLE, systemic lupus erythematosus; Sm, Smith uridine-1-5 ribonuclear protein antigen; SS-A, soluble substance-A.

The correlations among the autoantibody reactivities in patients with SLE were analysed and results are shown in the online supplementary table S1. Analysis of autoantibody reactivity to the nine antigens with reactivity frequency >5% showed several significant correlations (figure 3). Nucleosomes and histones were tightly correlated (r=1, p<0.001) although the sample sizes of reactive patients was low for nucleosomes n=3 compared with n=10 for histones. There was a negative correlation between Sm (n=6) and SSA (n=8) (r= −0.97, p<0.001). The most outstanding result was a strong negative correlation between anti-dsDNA (n=25) and anti-PCNA reactivity (n=33) (r=−1.0, p=0.008, figure 3). No patient expressed both anti-dsDNA and anti-PCNA autoantibody reactivity. Data from 9 patients with SLE (n=4 dsDNA reactive, and n=5 PCNA reactive) with follow-up testing showed that patients did not switch between the expression of anti-dsDNA versus anti-PCNA autoreactivity.

### Relating autoantibody reactivity profiles to clinical presentation in patients with SLE

**Figure 4:**
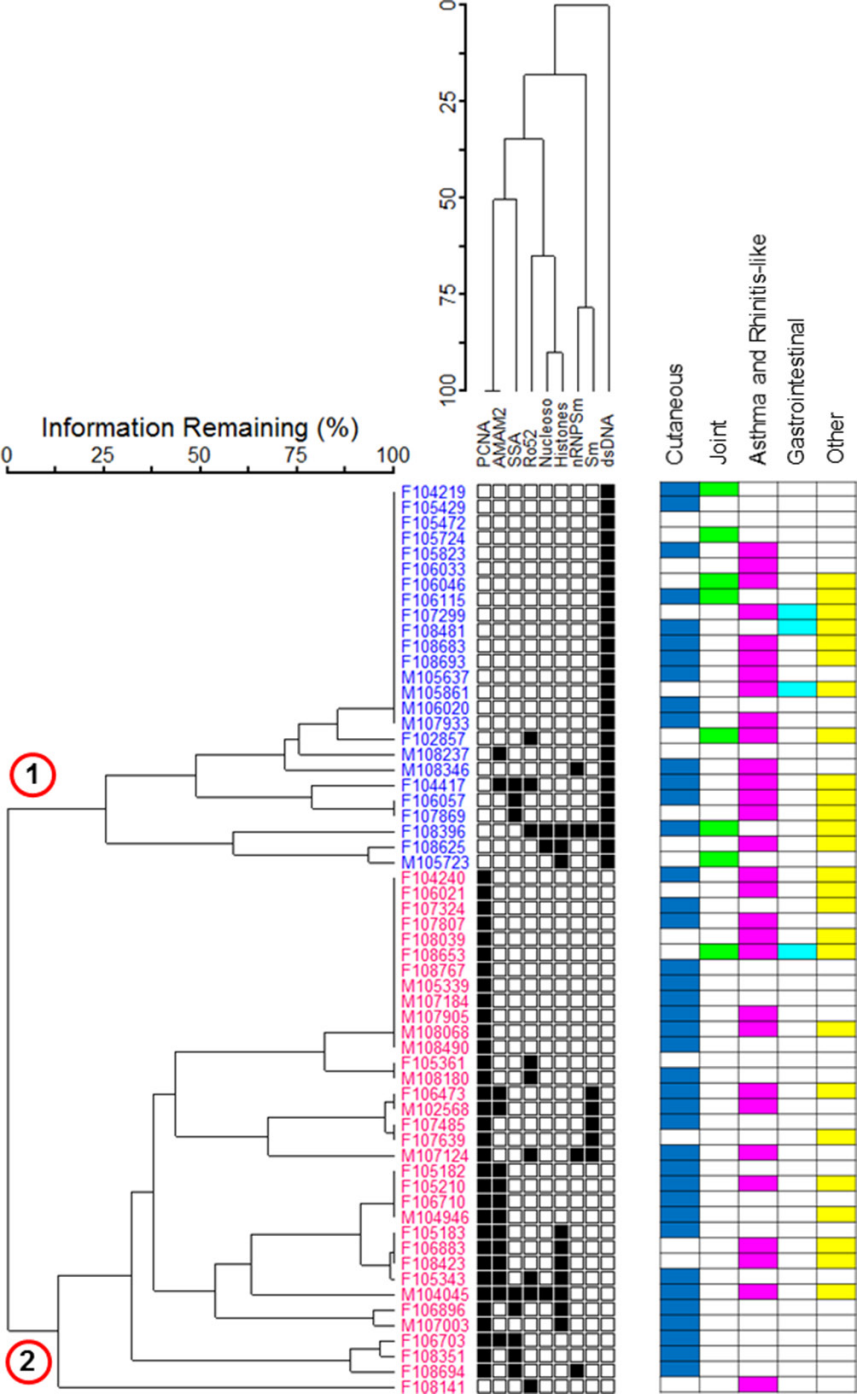
Two-way cluster analysis dendogram of patients with SLE (n=59, with two patients showing no ANA subtype reactivity excluded). Only the ANA subtypes with reactivity >5% reactivity frequency were included. The matrix of shaded squares represents the patient ×ANA reactivity matrix, while the dendrograms show the clustering. The dendrograms are scaled by Wishart’s objective function, expressed as the percentage of information remaining at each level of grouping.^22^ Each square represents the presence (black) and absence (white) of a reaction with a given biomarker. Two main clusters (designated 1 and 2) were identified. Matrix also indicates the presence of clinical symptoms with the colour key: blue=cutaneous symptoms; green=joint symptoms; purple=asthma and rhinitis-like symptoms; turquoise=gastrointestinal symptoms; yellow=other symptoms. AMA-M2, antimitochondrial antigen M2; ANA, antinulcear antibody; dsDNA, double-stranded DNA; nRNP/Sm, uridine 1-ribonuclear protein; PCNA, proliferating cell nuclear antigen; Ro-52, recombinant Ro-52; SLE, systemic lupus erythematosus; Sm, Smith uridine-1-5 ribonuclear protein antigen; SS-A, soluble substance-A.

Two-way cluster analysis identified two main groups: cluster 1 and cluster 2 (figure 4) based on the autoantibody reactivity profiles of patients with SLE. Cluster 1 comprised 25 patients; all were reactive against dsDNA but not the PCNA. Cluster 2 comprised 34 patients; 33 reactive against PCNA but not reactive against dsDNA and 1 that was not reactive against either dsDNA or PCNA. The expression of anti-dsDNA and anti-PCNA reactivity was mutually exclusive. More cluster 2 patients reacted against the mitochondrial antigen AMA-M2 compared with patients with SLE in cluster 1 (p=0.028).

Associations between cluster and clinical symptoms (online supplementary table S2) showed that cluster 1 was significantly associated with synovitis, arthritis or arthralgia (p=0.008; OR=12.8; 95% CI 1.41 to 595) (online supplementary table S3). Although patients in both clusters had cutaneous symptoms, patients in cluster 2 were significantly more likely to have only cutaneous symptoms (p=0.003; OR =9.1; 95% CI 1.71 to 88.6).

### Comparison of autoantibody reactivity profile of Black patients with SLE to reference ranges

**Figure 5:**
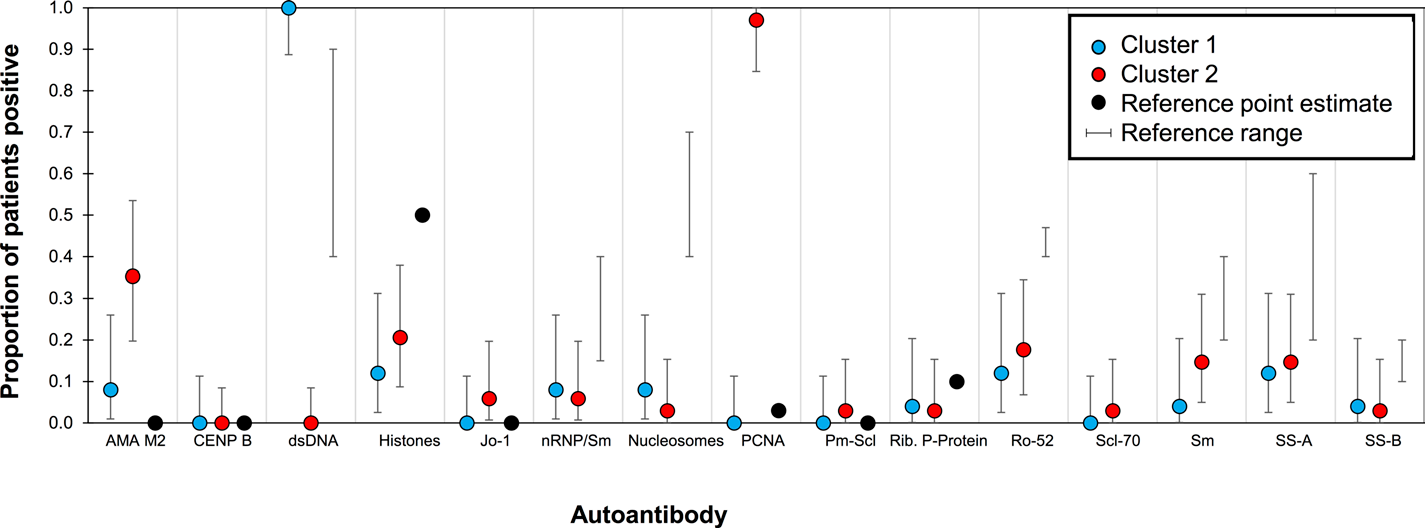
Comparison of reactivity frequency between patients with SLE and published reference ranges. Mean proportion of patients (n=61) positive for reactivity against the 15 ANA subtypes along with their exact 95% CIs. Data for the patients were divided into the two clusters identified in figure 4. Cluster 1 (blue) includes patients reacting primarily to dsDNA. Cluster 2 (red) includes patients reacting primarily with PCNA. Proportion of the reference population (recognised criteria^11 23–26^) is in black. Reference values: dots represent only a single value found for reference ANA frequency; bars represent lowest and highest in the range of values found for the reference. AMA-M2, antimitochondrial antigen M2; ANA, antinulcear antibody; CENP-B, centromere protein B; dsDNA, double-stranded DNA; Jo-1, cytoplasmic histidyl-tRNA synthetase antigen; nRNP/Sm, uridine 1-ribonuclear protein; PCNA, proliferating cell nuclear antigen; PM-Scl, polymyositis–sclerosis overlap antigen; Rib. P-protein, ribosomal P protein; Ro-52, recombinant Ro-52; SLE, systemic lupus erythematosus; Sm, Smith uridine-1-5 ribonuclear protein antigen; SS-A, soluble substance-A; SS-B, soluble substance-B.

The frequency or autoantibody reactivity directed against each antigen in the SLE patients was compared with the reference ranges published for SLE patients from different populations^11 23–26^ (figure 5, online supplementary table S4). The SLE patients were divided into the clusters identified in the cluster analysis. The frequency of PCNA, AMA-M2 and dsDNA reactivity was similar to the reference ranges for cluster one patients only. Patients in cluster two had a much higher reactivity frequency for both PCNA and AMA-M2 and a much lower reactivity frequency for dsDNA. Overall, the reactivity frequency patterns of Cluster one patients were similar though not identical to the published ranges, whereas Cluster two patients had a distinct autoantibody reactivity frequency pattern compared with the published ranges (figure 5).

10.1136/bmjgh-2017-000697.supp4 Supplementary data

## Discussion

Differences in SLE incidence and prevalence between different ethnic and racial groups have previously been documented in Americans in the United States^11 26 27^ and more recently in South Africans.^23^ Despite these ethnic differences, there is limited information regarding the frequency of antibody reactivity to different antigens among different races and ethnicities. Given the impact of both the environment and genetics on SLE incidence and possibly variable patterns of progression,^23^ there is need for SLE studies in Africa. Even in studies previously conducted in Black or African populations, for example, in Zimbabwe,^28 29^ Cameroon^30^ and South Africa,^23 31^ the nuclear antigens evaluated were often limited to dsDNA alone as the ACR recommended antigen associated with SLE. Here, the sero-reactivities of patients with SLE to a range of nuclear and mitochondrial antigens were characterised in a Black African population to inform diagnosis of SLE in Black patients resident in Africa.

As expected, significantly more patients with SLE were ANA reactive as assessed by immunoblot analysis of ANA subtype reactivity, compared with the controls diagnosed negative for any connective tissue or allergic disease by clinical history and examination. The antimitochondrial antigen AMA-2 is associated with Primary Biliary Cirrhosis is not routinely determined in SLE. It was measured in this study because it was part of the manufacturer’s autoantibody strip. Nonetheless, in this study, the high frequency of anti-AMA-M2 reactivity in both the SLE-negative participants and some patients with SLE is intriguing. Although some tropical infectious diseases, for example, malaria, hepatitis B and tuberculosis, induce ANA reactivity,^32^ in this group, no participants (patients or controls) were diagnosed with these infections. Two participants clinically diagnosed patients with SLE did not show any ANA subtype reactivity. This is not unusual since autoreactivity can mirror disease severity and SLE clinical presentation in the absence of ANA reactivity is known to occur in about 5% of patients with SLE.^33^ In view of the profile and patient scope of the clinic, it was not surprising that a number of the patients with SLE had allergy-related symptoms in addition to having SLE, however sample sizes of this group were too small to conduct any statistical analyses.

Correlation analyses showed different relationships between reactivities against different nuclear antigens. The outstanding novel results, the strong negative correlation between dsDNA, the hallmark SLE autoantigen^34^ and PCNA with the groups of patients reacting against these two antigens, were mutually exclusive.

Forty-one per cent of the patients with SLE in the study were reactive against the dsDNA antigen, a frequency within the published range of 40%–90%.^11^ The only previous ANA study in Zimbabwe showed a 100% dsDNA reactivity frequency among patients with SLE, but that study tested just seven Black patients.^29^ The reactivity profile of the dsDNA-reactive patients with SLE in this present study was largely similar to frequency ranges published for other African or African American populations, including those from the large retrospective study of Tunisians,^35^ South Africans^23^ and African Americans^26^ and it was consistent with the 2012 SLICC criteria^9^ for SLE. Fifty-four percent of the patients with SLE were negative for reactivity against dsDNA, but were reactive against PCNA. This is notably higher than 1%–3%^36^ or 5%–10%^34^ PCNA frequencies reported in the literature. The age ranges between the dsDNA and PCNA reactive groups were similar so that age was not a determinant of ANA reactivity pattern. In addition, none of the nine patients who followed up two to four times during the 4-year study period switched their autoantibody reactivity between these two antigens.

PCNA reactivity has been reported as expressed in patients with SLE with a very high specificity (99%).^36–38^ It is remarkable that while the literature reports the presence of anti-PCNA reactivity in only a handful of patients,^34 36^ the proportion reported in this study was considerably higher.

Further analysis showed that patients with SLE fell into one of two distinct clinical clusters. The first subgroup (44% of these patients) generally follows the revised ACR and SLICC SLE classifications with ANA subtype profile anchored by anti-dsDNA reactivity. This group was 13 times more likely to present with synovitis and tended to have a severe clinical course with frequent mucosal, joint and renal involvement. It is interesting to note that the published studies in Black African patients with SLE that have reported a high frequency of anti-dsDNA antibodies, for example, 67%, 100% and 74% in Black South Africans,^31^ Zimbabweans^29^ and Cameroonians^30^ respectively, have also reported high levels of joint clinical symptoms (arthritis), that is, 62%, 81% and 64% of the population, respectively. It is therefore possible that these studies are reporting the form of SLE present in the dsDNA-reactive patients, that is, those in cluster 1.

The second group (56%) of patients with SLE had an autoantibody reactivity profile characterised by anti-PCNA. An associated antigen in this patient group was anti-AMA-M2 which is a characteristic of primary biliary cirrhosis.^39–41^ The patients in this study did not have any clinical or laboratory features of primary biliary cirrhosis or autoimmune hepatitis. Their liver enzymes alanine transaminase, aspartate aminotransferase, alkaline phosphatase and albumin were normal. The PCNA subtype reactivity profile has not previously been described at such a high frequency in the literature nor have the PCNA reactivity frequencies and association with cutaneous symptoms previously been reported in African populations. This subgroup was nine times more likely to present with cutaneous symptoms including pruritic, annular or papulosquamous dermatoses that healed without scarring as seen in some forms of cutaneous lupus erythematosus (CLE).^42^ The coexpression of anti-PCNA and AMA-M2 reactivity may be indicative of a subgroup of patients with the cutaneous variant of lupus and warrants further investigation. There is need to explore whether genetic variants in the HLA region explain the existence of the two identified clusters. There were other associations between, for example, histones and Sm in patients with SLE positive for anti-PCNA reactivity, but sample sizes were too small to make any conclusive interpretations. These relationships and their biological meaning require further explorations in larger sample sizes.

A recent initiative to inform SLE management in African patients (the African Lupus Genetics Network) identified underdiagnosis/delayed diagnosis as challenges to care delivery.^4^ A Zimbabwean study reported that inclusion of the ANA reactivity (dsDNA and Sm) increased the sensitivity of the SLE diagnostic criteria.^28^ Thus, the inclusion of PCNA may further inform these diagnostic criteria and improve sensitivity. Given these data, a prospective study of patients with SLE will allow more detailed characterisation of the immunology and molecular features of the Zimbabwean patients. This will overcome the constraints/biases of retrospective studies.

In conclusion, our study suggests that there exists a large subgroup (~50%) of Southern African patients whose ANA subtype reactivity is directed against the PCNA. This profile differs from the ACR and SLICC classification of SLE which only recommend testing for the anti-dsDNA and anti-Sm antibodies. The findings suggest that a reliance on the ACR laboratory criteria for SLE diagnosis may not be adequate for all patient groups residing in Africa. Furthermore, anti-PCNA antibodies may be a marker for cutaneous variants of SLE. Our study shows a need to (i) widen the panel of diagnostic antinuclear and anti-mitochondrial antigens used in African patients, (ii) further refine the predictive values of the ANA reactivity to different nuclear and mitochondrial antigens in order to differentiate SLE syndromes in African populations, and (iii) consider anti-PCNA reactivity, which has so far been largely excluded in Africa and elsewhere,^43^ as a diagnostic marker for patients with SLE.
