# Supplementary material for: Evidence of a distinct group of Black African patients with systemic lupus erythematosus
Source: BMJ Glob Health. 2018 Sep 16;3(5):e000697. doi: 10.1136/bmjgh-2017-000697 (PMC6144901; doi:10.1136/bmjgh-2017-000697)
Supplement: Supplementary data [file bmjgh-2017-000697supp001.pdf]

	**AMA M2**	**dsDNA**	**Histones**	**Jo-1**	**nRNPS/Sm**	**Nucleosomes**	**PCNA**	**PM-Scl**	**Rib. P-Protein**	**Ro-52**	**Scl-70**	**Sm**	**SS-A**	**SS-B**
**AMA M2**	1.0000	-0.5386	0.4930	-0.9325	-0.9716	0.1518	0.6052	-0.9382	-0.9325	0.2029	0.9821	0.1790	0.2659	0.3317
**dsDNA**	-0.5386	1.0000	-0.1915	-0.9504	0.1171	0.3096	-0.9992	-0.9504	0.1021	-0.1279	-0.9504	-0.3846	-0.0556	0.1021
**Histones**	0.4930	-0.1915	1.0000	-0.9238	0.1599	0.9999	0.2695	-0.9495	0.9873	0.3664	-0.9495	0.0062	0.1937	-0.9238
**Jo-1**	-0.9325	-0.9504	-0.9238	1.0000	0.6784	-0.8762	0.9556	-0.8362	-0.8734	-0.9224	-0.8362	-0.9249	0.9990	-0.8734
**nRNPS/Sm**	-0.9716	0.1171	0.1599	0.6784	1.0000	0.5520	-0.0529	-0.8771	0.6784	0.5393	-0.8771	0.6663	0.2378	-0.9564
**Nucleosomes**	0.1518	0.3096	0.9999	-0.8762	0.5520	1.0000	-0.2521	-0.8863	0.7437	0.6706	-0.8863	0.4338	0.3447	-0.8762
**PCNA**	0.6052	-0.9992	0.2695	0.9556	-0.0529	-0.2521	1.0000	0.9556	-0.0461	0.0235	0.9556	0.4435	0.1316	-0.0461
**PM-Scl**	-0.9382	-0.9504	-0.9495	-0.8362	-0.8771	-0.8863	0.9556	1.0000	-0.8362	-0.9588	-0.8361	-0.8813	-0.8895	-0.8362
**Rib. P-Protein**	-0.9325	0.1021	0.9873	-0.8734	0.6784	0.7437	-0.0461	-0.8362	1.0000	0.4694	-0.8362	0.5793	-0.9216	-0.8734
**Ro-52**	0.2029	-0.1279	0.3664	-0.9224	0.5393	0.6706	0.0235	-0.9588	0.4694	1.0000	-0.9588	0.3639	0.2395	0.4694
**Scl-70**	0.9821	-0.9504	-0.9495	-0.8362	-0.8771	-0.8863	0.9556	-0.8361	-0.8362	-0.9588	1.0000	-0.8813	0.9848	-0.8362
**Sm**	0.1790	-0.3846	0.0062	-0.9249	0.6663	0.4338	0.4435	-0.8813	0.5793	0.3639	-0.8813	1.0000	-0.9689	-0.9249
**SS-A**	0.2659	-0.0556	0.1937	0.9990	0.2378	0.3447	0.1316	-0.8895	-0.9216	0.2395	0.9848	-0.9689	1.0000	0.5027
**SS-B**	0.3317	0.1021	-0.9238	-0.8734	-0.9564	-0.8762	-0.0461	-0.8362	-0.8734	0.4694	-0.8362	-0.9249	0.5027	1.0000
