# Supplementary material for: Evidence of a distinct group of Black African patients with systemic lupus erythematosus
Source: BMJ Glob Health. 2018 Sep 16;3(5):e000697. doi: 10.1136/bmjgh-2017-000697 (PMC6144901; doi:10.1136/bmjgh-2017-000697)
Supplement: Supplementary data [file bmjgh-2017-000697supp002.pdf]

Table S2A: Grouping of clinical symptoms used in analyses

cutaneous	Joint	Asthma and Rhinitis like	Gastrointestinal	Other
acne	joint pain	tightness of the chest	abdominal discomfort	sore tongue
rash on neck and face	knee swelling	wheezing	oesophageal reflux	red eyes
dermatitis	painful knees and ankles	cough	abdominal irritation	discharge from eyes
eczema	neck pain	shortness of breath	stomach bloating	headache
hair loss	joint swelling	constant phlegm		dizziness
alopecia	hip/back-ache	breathing difficulties		numbness of fingers and toes
fungal erruptions		dry irritating cough		boils on lips
thigh itchiness		asthma		otitis
generalised body rash		continuous flu		palpitations
rash on exposure to the sun		block nose		hot feet
itchines sof skin		itchy (eyes, palate, ears)		fatigue
generalised uticaria				visual problems
itchy hives				
facial rash				
dry skin				
perioral hyperpigmentation				
pruritus				
angioedema				
swollen lips				
swelling of the skin				
swelling of face				
generalised swelling				
