# Supplementary material for: Evidence of a distinct group of Black African patients with systemic lupus erythematosus
Source: BMJ Glob Health. 2018 Sep 16;3(5):e000697. doi: 10.1136/bmjgh-2017-000697 (PMC6144901; doi:10.1136/bmjgh-2017-000697)
Supplement: Supplementary data [file bmjgh-2017-000697supp003.pdf]

Table S3. Table to accompany Figure 2. Number and proportion of participants diagnosed with Systemic Lupus Erythematosus (SLE) (n=61) and study control patients (n=100) who are reactive against each antinuclear antigens.

**Autoimmunity biomarker**	**Controls**			**SLE**		
	**positive**	**Total**	**proportion**	**positive**	**Total**	**proportion**
AMA-M2	8	100	0.080	14	61	0.230
CENP-B	0	100	0.000	0	61	0.000
ds-DNA	0	100	0.000	27	61	0.443
Histones	0	100	0.000	10	61	0.164
Jo-1	2	100	0.010	2	61	0.033
nRNP/Sm	2	100	0.020	4	61	0.066
Nucleosomes	0	100	0.000	3	61	0.049
PCNA	0	100	0.000	34	61	0.557
PM-Scl	1	100	0.010	1	61	0.016
Rib P-protein	0	100	0.000	2	61	0.033
Scl-70	0	100	0.000	1	61	0.016
Sm	3	100	0.030	6	61	0.098
SS-A/Ro	0	100	0.000	8	61	0.131
SS-A Ro-52	2	100	0.020	9	61	0.148
SS-B/La	0	100	0.000	2	61	0.033
