# Supplementary material for: Evidence of a distinct group of Black African patients with systemic lupus erythematosus
Source: BMJ Glob Health. 2018 Sep 16;3(5):e000697. doi: 10.1136/bmjgh-2017-000697 (PMC6144901; doi:10.1136/bmjgh-2017-000697)
Supplement: Supplementary data [file bmjgh-2017-000697supp004.pdf]

Table S4. Table to accompany Figure 5. Mean proportion of patients (n=61) reactive against the 15 antigens along with their exact 95% confidence intervals. Data for the patients were divided into the two clusters identified in Figure 4. Cluster 1 (blue) includes patients reacting primarily to ds-DNA. Cluster 2 (red) includes patients reacting primarily against PCNA. Proportion of the reference population (recognised criteria [10,19,20]) is in black. Reference values are either a single value or a range.

| Autoimmunity biomarker | Cluster 1 | Cluster 2 | Reference |
|---|---|---|---|
| AMA-M2 | 0.080 (0.010 - 0.260) | 0.353 (0.197 – 0.535) | 0 |
| CENP-B | 0.000 (0.000 - 0.113) | 0.000 (0.000 – 0.084) | 0 |
| ds-DNA | 1.000 (0.887-1.00) | 0.000 (0.000 – 0.084) | 40-90 |
| Histones | 0.120 (0.025 – 0.312) | 0.206 (0.087 – 0.379) | 50 |
| Jo-1 | 0.000 (0.000- 0.113) | 0.059 (0.007 – 0.197) | 0 |
| nRNP/Sm | 0.080 (0.010 - 0.260) | 0.059 (0.007 – 0.197) | 15-40 |
| Nucleosomes | 0.080 (0.010 - 0.260) | 0.029 (0.001 – 0.153) | 40-70 |
| PCNA | 0.000 (0.000 - 0.113) | 0.971 (0.847 – 0.999) | 3 |
| PM-Scl | 0.000(0.000 - 0.113) | 0.029 (0.001 – 0.153) | 0 |
| Rib P-protein | 0.040 (0.001-0.204) | 0.029 (0.001 – 0.153) | 10 |
| Scl-70 | 0.000 (0.000 - 0.113) | 0.029 (0.001 – 0.153) | 0 |
| Sm | 0.040 (0.001-0.204) | 0.147 (0.050 – 0.311) | 20-40 |
| SS-A/Ro | 0.120 (0.025 – 0.312) | 0.147 (0.050 – 0.311) | 20-60 |
| SS-A Ro-52 | 0.120 (0.025 – 0.312) | 0.177 (0.068 – 0.345) | 40-47 |
| SS-B/La | 0.040 (0.001-0.204) | 0.029 (0.001 – 0.153) | 10-20 |
